# Why are primary school children overweight and obese? A cross sectional study undertaken in Kinondoni district, Dar-es-salaam

**DOI:** 10.1186/s12889-015-2598-0

**Published:** 2015-12-21

**Authors:** Sijenunu A. Mwaikambo, Germana H. Leyna, Japhet Killewo, Azma Simba, Thandi Puoane

**Affiliations:** Ministry of Health and Social Welfare, Dar es Salaam, United Republic of Tanzania; School of Public Health, Muhimbili University of Health and Allied Sciences, Dar es Salaam, United Republic of Tanzania; School of Public Health, University of the Western Cape, Bellville, South Africa

**Keywords:** Overweight, Obesity, Children, School, Dar es Salaam

## Abstract

**Background:**

The world is experiencing an alarming increase in prevalence of childhood obesity. Despite this trend little is known about determinants of childhood obesity in Tanzania. A cross sectional study determined the prevalence and factors associated with overweight and obesity in 1722 children aged 7–14 years (10.9 ± 1.74) attending primary schools in Dar es Salaam.

**Methods:**

Six public and four private schools were systemically selected from a total of 227 primary schools. Anthropometric measurements (weight and height) were collected using a standard protocol and Body Mass Index (BMI) was calculated. Interviews collected demographic characteristics and lifestyle factors. Multiple logistic regression test was used to assess the influence of independent variables on overweight and obesity while controlling for confounding factors. The level of significance was set at α = 5 %.

**Results:**

Of 1, 722 children 10.2 % were overweight and 4.5 % were obese. Overweight and obesity was higher in boys (14.9 %) than girls (14.5 %), higher in children attending private schools (27.7 %) than public schools (5.9 %). Children who walked to and from school were less likely to be overweight or obese than those who used vehicles (AOR = 0.5; 95%CI: 0.3–0.6; *p* < 0.001). Those who used private cars or school buses were more likely to be overweight or obese than those who used public transport (AOR = 2.9; 95%CI: 0.2–0.7; *p* < 0.05). Computer/video game use were associated with increased risk of overweight and obesity (AOR = 1.6; 95%CI: 1.1–2.3; *p* = 0.03). Lunch provided by schools was associated with increased risk of overweight or obese (AOR = 6.4, 95 % CI = 4.2–9.6, *p* < 0.001).

**Conclusions:**

The findings of this study identified a number of behavioural and dietary factors that are related to overweight and obesity. Parents and teachers should encourage children to be physically active by limiting screen time and promoting active transport to and from school to promote health and reduce obesity. Ministry of education needs to formulate/enforce policies that encourage physical activities for school children and regulate quality of foods provided to children at schools.

## Background

The world is experiencing an alarming increase in prevalence of childhood overweight and obesity [1]. The worldwide prevalence of childhood overweight and obesity increased dramatically from 4.2 % in 1990 to 6.7 % in 2010, [2]. Globally a total of 42 million children under the age of five were estimated to be overweight or obese in 2010 and 92 million were at risk of overweight. A high proportion of this burden (35 million children) was borne by developing countries [2]. The estimated prevalence of childhood overweight and obesity in Africa in 2010 was 8.5 % [2], while that of Asia was 4.9 % and Latin America and Caribbean was 6.9 %. This trend is expected to reach 9.1 % (60 million) worldwide and 12.7 % in Africa in 2020 [2].

Studies conducted in many parts of the world have shown physical inactivity including lack of participation in sports activities at school or at home, and less active mode of transport to and from school to be associated with increased risk of overweight and obesity among children [3–5]. Diets high in saturated fats, sugars and refined starch have also been shown to contribute to the increasing overweight and obesity among children and adults [6, 7]. Furthermore, the increase in time spent watching television and playing electronic games partially contributes to the increasing rates of obesity among children [8–14].

However, there is limited information on the magnitude of overweight and obesity in Tanzania given the increase in urbanization, demographic and nutrition transition in the country, particularly in urban areas. The few studies that have been reported have been carried out in rural populations [15], used only the World Health Organization’s (WHO) BMI for age percentile and body fat mass measurements [15] or estimated their samples size using an expected prevalence of obesity among women of reproductive age making precision of the sample size estimate questionable [16]. In the current study we used the International Obesity Task Force (IOTF) cut off points for classifying children as overweight or obese to estimate the prevalence of overweight and obesity in Tanzanian school children. The IOTF BMI classification was found to have excellent specificity and sensitivity for overweight cut off points when compared to United Kingdom data and body impedance measure of total fat [17]. In addition, this method was developed using International data, thus allows international comparisons of overweight and obesity in children.

## Methods

### Study design, setting and population

This was a Cross sectional study of children aged 7–14 years attending primary schools in Kinondoni District Council, Dar es Salaam. This study was conducted in Kinondoni municipality, which is among the three municipalities of Dar es Salaam city; the commercial capital of Tanzania and home for 4.1 % of the total Tanzania mainland population [18]. A majority of the people in the urban part of the municipality engage in self-employment activities such as trading, fishing and small-scale manufacturing in the informal sector [19].

### Sample size & sampling

A total of 1, 793 children were estimated using a cluster sampling formula [20]. Using systematic random sampling, six public and four private schools were selected from a total of 227 primary schools (of which 137 were public and 90 were private). The sampling interval for both schools was 23. A total of 180 children were recruited from each of the sampled schools. Systematic random sampling was used to select classes that make up 180 children and all children in a sampled class were included in the study.

### Data collection

A structured questionnaire administered by trained research assistants was used to collect demographic characteristics and lifestyle factors (diet and physical activity) from all children whose parents gave consent and the children assented to participate in the study. The collected demographic data included age, sex, residence, number of siblings, parents’ education and occupation. Physical activity data collected were mode of transport to and from school; and television/computer/video game use. Regarding television/computer/video game use, children were asked if they usually used these gadgets where “usually” was defined as at least once every week. Children who reported to “usually” use were asked about days per week and hours per day spent and hours used per week was calculated. The collected diet data included use of full sugar drinks, diet soft drinks, fruit juice, fried food, starch foods, fruits, vegetables, taking breakfast, time heaviest meal of the day taken, and dinner time. Also children were asked if they were given money to buy food at school, the amount of money given and type of food they buy, if they are given lunch at school, type of food given and if they carry lunch boxes to school.

For each child weight in kilograms (kg) was collected using a digital weighing scale that was regularly calibrated to a known weight. Weight measurements were rounded to one decimal place. A Shorr portable measuring board model 420 (Olney, MD 20822, USA) was used to measure height in metres (m). Two measurements were taken and the average was recorded. All measurements were done with children dressed in light clothing with no shoes (Reference Stepwise methodology) [21]. BMI (kg/m^2^) was calculated by dividing the weight (kg) by the squared height (m^2^). Each child was classified as overweight or obese according to the International Obesity Task Force (IOTF) BMI cut off-points for defining overweight and obesity in children between 2 and 18 years. Children with BMI corresponding to a projected BMI of 25 to <30 and greater or equal to 30 at age 18 were categorized as overweight and obese respectively. All children with a projected BMI of below 25 at age 18 were categorized as normal weight or underweight [17].

### Ethical considerations

Ethical clearance to conduct the study was granted by the Muhimbili University of Health and Allied Sciences (MUHAS), Research and Publication Ethical Committee. Permission was also sought from the District Executive Director and head teachers of schools that participated in the study. Written consent for children’s participation in the study was sought from school authority and parents. All children provided a verbal assent to participate.

### Statistical analysis

Data was analysed using Epi Info software version 3.5.1. Continuous variables were summarized using means and standard deviations and categorical data using frequencies and percentages. Univariate analysis was done for demographic and lifestyle factors. Differences in levels of exposure were statistically tested using Student’s *t* test and Chi square test for continuous and categorical variables, respectively. Odds ratios were estimated to ascertain association between dependent variables (overweight, obesity) and key explanatory variables. Variables were added to the multiple logistic regression models if they had a correlation with BMI at *p* < 0.2. Adjusted Odds ratios and their 95 % confidence intervals were reported. All analysis was two-tailed at α = 5 %.

## Results

A total of 1722 children aged 7–14 yeas (10.9 ± 1.74) participated in the study, a response rate of 96 %. Table 1 shows background characteristics of children who participated in the study and their Body Mass Index. Of 1722 children who participated, 175 (10.2 %) were overweight and 78 (4.5 %) were obese. Among the girls 98 (10.4 %) were overweight and 39 (4.1 %) were obese while 77 (9.9 %) and 39 (5 %) of the boys were overweight and obese respectively. Overweight and obesity was more prevalent in children who attended private primary schools, 18.2 and 9.5 % respectively (*p* < 0.001). Prevalence of overweight and obesity was higher among participants who had 1–3 siblings 181 (16.8 %) (p 0.001); whose parents had secondary and post-secondary education, 157 (17.9 %) and 164 (20.2 %) of those whose fathers and mothers had secondary and post secondary education respectively (*p* = 0.00); used private cars/school buses to and from school 139 (28.2 %) (*p* = 0.0001), often watched television 228 (15.7 %), (*p* < 0.001), often played computers or video games 132 (22.0 %) (*p* = <0.0001). The prevalence of overweight and obesity was lower among children who reported usually walking to and from school, 103 (9.3 %) compared to those who reported did not, 150 (24.6 %) (p 0.00) as shown in Table 1.

**Table 1:** Background characteristics and body mass index of children

Characteristics	No of subjects	Normal and underweight	Overweight (BMI 25– < 30)	Obese (BMI > =30)	Overweight/obese	p-value*
	(*n* = 1722)	(*n* = 1469)	(*n* = 175)	(*n* = 78)	(*n* = 253)	
		n (%)	n (%)	n (%)	n (%)	
Gender						
Girls	943	806 (85.5)	98 (10.4)	39 (4.1)	137 (14.5)	0.82
Boys	779	663 (85.1)	77 (9.9)	39 (5.0)	116 (14.9)	
Age group (Yrs)						
7–10 (9 ± 0.97)	639	536 (83.9)	69 (10.8)	34 (5.3)	103 (16.1)	0.21
11–14 (12 ± 0.93)	1083	933 (86.1)	106 (9.8)	44 (4.1)	150 (13.9)	
Area of residence						
Kinondoni	1684	1439 (85.5)	169 (10.0)	76 (4.5)	245 (14.5)	0.26
Other	38	30 (78.9)	6 (15.8)	2 (5.2)	8 (21.0)	
School type						
Public	1030	969 (94.1)	49 (4.8)	12 (1.2)	61 (6.0)	<0.001
Private	692	500 (72.3)	126 (18.2)	66 (9.5)	192 (27.7)	
Number of siblings						
0–3	1073	892 (83.1)	126 (11.7)	55 (5.1)	181 (16.8)	0.001
4 and more	649	577 (88.9)	49 (7.6)	23 (3.5)	72 (11.1)	
Father’s level of education						
Secondary/post secondary	879	722 (82.1)	113 (12.9)	44 (5.0)	157 (17.9)	<0.001
Primary/no formal education	279	259 (92.8)	14 (5.0)	6 (2.2)	20 (7.2)	
Don’t know	564	488 (86.5)	48 (8.5)	28 (4.9)	76 (13.5)	
Mother’s level of education						
Secondary/post secondary	813	649 (79.8)	115 (14.1)	49 (6.1)	164 (20.2)	<0.001
Primary/formal education	413	386 (93.5)	20 (4.8)	7 (1.7)	27 (6.5)	
Don’t know	496	434 (87.5)	40 (8.1)	22 (4.4)	62 (12.5)	
Mode of transport to and from school: Walking						
Yes	1112	1009 (90.7)	79 (7.1)	24 (2.2)	103 (9.3)	<0.001
No	610	460 (75.4)	96 (15.7)	54 (8.9)	150 (24.6)	
Mode of transport to and from school: Private cars and public transport						
Private car or school bus	493	354 (71.8)	87 (17.6)	52 (10.5)	139 (28.2)	<0.0001
Public transport	117	106 (90.6)	9 (7.7)	2 (1.7)	11 (9.4)	
Usually watch television						
Yes	1449	1221 (84.3)	154 (10.6)	74 (5.1)	228 (15.7)	<0.001
No	273	248 (90.8)	21 (7.7)	4 (1.5)	25 (9.2)	
Usually use computers						
Yes	576	440 (76.4)	91 (15.8)	45 (7.8)	136 (23.6)	<0.0001
No	1146	1029 (89.8)	84 (7.3)	33 (2.9)	117 (10.2)	
Usually play video games						
Yes	600	468 (78.0)	87 (14.5)	45 (7.5)	132 (22.0)	<0.0001
No	1122	1001 (89.2)	88 (7.8)	33 (2.9)	121 (10.7)	
Given money to buy food at school						
Yes	1232	1087 (88.2)	101 (8.2)	44 (3.6)	145 (11.8)	<0.0001
No	490	382 (77.9)	74 (15.1)	34 (7.0)	108 (22.1)	
Amount of money given						
<=1000Tshs (<=0.5USD)	1144	1018 (88.9)	88 (7.7)	38 (3.3)	126 (11.0)	0.008
>1000Tshs (>0.5USD)	88	70 (79.5)	12 (13.6)	6 (6.8)	18 (20.5)	
Given food to take to school						
Yes	242	194 (80.2)	31 (12.8)	17 (7.0)	48 (19.8)	0.02
No	1480	1275 (86.1)	144 (9.7)	61 (4.1)	205 (13.9)	
Given food at school						
Yes	692	500 (72.3)	126 (18.2)	66 (9.5)	192 (27.7)	<0.001
No	1030	969 (94.1)	49 (4.8)	12 (1.2)	61 (5.9)	
Drink light sugar soft drinks						
Yes	829	683 (82.4)	98 (11.8)	48 (5.8)	146 (17.6)	0.001
No	893	786 (88.0)	77 (8.6)	30 (3.4)	107 (11.9)	
Dinner time						
<=7:00 PM	1536	1331 (86.7)	146 (9.5)	59 (3.8)	205 (13.3)	0.06
>7:00 PM	186	152 (81.7)	22 (11.8)	12 (6.5)	34 (18.3)	
Usually eat breakfast before going to school						
Yes	1198	1023 (85.4)	127 (10.6)	48 (4.0)	175 (14.6)	0.87
No	524	446 (85.1)	48 (9.2)	30 (5.7)	78 (14.9)	
Drink full sugar soft drinks						
Yes	1356	1157 (85.3)	137 (10.1)	62 (4.6)	199 (14.7)	0.96
No	366	312 (85.2)	38 (10.4)	16 (4.4)	54 (14.8)	
Usually eat vegetables						
Yes	1675	1429 (85.3)	170 (10.1)	76 (4.5)	246 (14.7)	0.97
No	47	40 (85.1)	5 (10.6)	2 (4.3)	7 (14.9)	
Usually drink fruit juice						
Yes	1612	1368 (84.9)	169 (10.5)	75 (4.7)	244 (15.1)	<0.001
No	110	101 (91.8)	6 (5.5)	3 (2.7)	9 (8.2)	
Usually eat fruits						
Yes	1693	1443 (85.2)	174 (10.3)	76 (4.5)	250 (14.8)	0.49
No	29	26 (89.7)	1 (3.4)	2 (6.9)	3 (10.3)	
Usually eat fried food						
Yes	1623	1389 (85.6)	163 (10.0)	71 (4.4)	234 (14.4)	0.19
No	99	80 (80.8)	12 (12.1)	7 (7.1)	19 (19.2)	
Usually eat starch foods						
Yes	1647	1407 (85.4)	169 (10.3)	71 (4.3)	240 (14.6)	0.52
No	75	62 (82.7)	6 (8.0)	7 (9.3)	13 (17.3)	

*p value corresponds to a comparison of prevalence of overweight/obese children among different groups

### Factors associated with overweight and obesity

Children who reported living with three or less siblings were more likely to be overweight or obese than children living with more siblings (COR = 1.6, 95 % CI = 1.2–2.2, p 0.001). Children whose fathers had secondary or post secondary education were more likely to be overweight or obese than children whose fathers had no formal education or had primary education (COR = 2.8, 95 % CI = 1.8–4.5, *p* < 0.001). Similarly children whose mothers had secondary or post secondary education were more likely to be overweight or obese than children whose mothers had no formal or primary school education (COR = 3.6, 95 % CI = 2.4–5.6, *p* < 0. 0001).

Children who reported walking to and from school were less likely to be either overweight or obese than those who reported using other means of transport (COR = 0.3, 95 % CI = 0.2–0.4, *p* < 0. 0001). Children who reported using school bus or private cars were 4.2 times more likely to be overweight or obese than those who reported using public transport (COR = 4.2, 95 % CI = 1.9–8.9, *p* < 0.0001).

Children who reported watching television were 1.9 times more likely to be overweight or obese than those who did not normally watch television. Children who reported to normally watch television (*n* = 1449), reported to watch for 0.2–105 h per week (8 h ± 10.3). Those who watched television for more than 14 h per week were more likely to be overweight or obese than those who reported watching for 14 h or less per week (COR = 1.97, 95 % CI = 1.4–2.9, p 0.0003). Mean time spent watching television per week was almost the same for girls (7.9 h ± 10.89) and boys (8.2 h ± 9.54). Similarly mean time for age group 11–14 years (7.49 ± 9.44) was almost the same as age group 7–10 years (8.87 ± 11.51).

Computer use was reported by 576 (33.4 %) children. Mean time of using computers was 5.4 h per week ± 7.96. Mean time for girls was 5.75 ± 8.74 and for boys was 4.97 ± 7.11. Mean time for age group 11–14 years was 4.71 ± 7.02 and for age group 7–10 years was 6.28 ± 9.07. Children who reported often using computers for more than six hours per week were more likely to be overweight or obese than those who reported spending less time on computers (COR = 1.6, 95 % CI = 1.1–2.5, *p* < 0. 05). Similarly, respondents who reported playing video/computer games were more likely to be overweight or obese than children who did not often play video games (COR = 2.3, 95 % CI = 1.8–3.1, *p* < 0.001).

After adjusting for gender, age and school type, having 1–3 siblings, parents with secondary or post secondary education, using private cars or school bus to and from school, and using computers were found to be associated with increased risk of overweight and obesity as shown in Table 2. Walking to and from school was found to be protective against overweight and obesity.

**Table 2:** Factors associated with child overweight or obesity

Variable	Categories	Overweight/Obese	
		Model 1	Model 2
		COR (95 % CI)	AOR (95%CI)
Number of siblings	0–3	1.6 (1.2–2.2)*	1.4 (1.5–1.9)*
	4 and more	1.0	1.0
Father’s level of education	Secondary or post secondary	2.8 (1.8–4.5)**	2.8 (1.7–4.6)**
	Primary or no formal education	1.0	1.0
Mother’s level of education	Secondary or post secondary	3.6 (2.4–5.6)***	2.74 (1.5–4.9)**
	Primary or no formal education	1.0	1.0
Mode of transport to and from school: Walking	Yes	0.3 (0.2–0.4)***	0.5 (0.3–0.6)**
	No	1.0	1.0
Mode of transport to and from school: Other modes of transport	Private car or school bus	4.2 (1.9–8.9)***	2.9 (0.2–0.7)*
	Public transport	1.0	1.0
Usually watch television	Yes	1.9 (1.2–2.9)*	1.3 (0.8–2.1)
	No	1.0	1.0
Time used to watch television per week	Above 14 h	2.0 (1.4–2.9)**	1.6 (1.0–1.5)
	14 h and less	1.0	1.0
Usually use computers	Yes	2.7 (2.1–3.6)***	1.6 (1.1–2.3)*
	No	1.0	1.0
Time used on computers per week	>6 h	1.6 (1.1–2.5)*	1.5 (1.1–1.4)
	6 h or less	1.0	1.0
Usually play video games	Yes	2.3 (1.8–3.1)***	1.2 (0.8–1.7)
	No	1.0	1.0

*p-value < 0.05

**p-value < 0.001

***p-value < 0.0001

### Overweight and obesity and dietary practices

Table 3 shows that children who were given money to buy food at school were less likely to be overweight or obese than those not usually given money (COR = 0.5, 95 % CI = 0.4–0.6, *p* < 0.0001). However among those given money, children who reported to be given 1000 T.shs (0.5 USD) and less per day were less likely to be overweight or obese than those given more than 1000 T.shs per day (COR = 0.49, 95 % CI = 0.3–0.9, *p* < 0.001).

**Table 3:** Association between children’s dietary practices and overweight or obesity

Dietary practices	Categories	Overweight/Obese	
		Model 1	Model 2
		COR (95 % CI)	AOR (95%CI)
Given money to buy food at school	Yes	0.5 (0.4–0.6)***	0.7 (0.5–0.9)*
	No	1.0	1.0
Amount of money given	<=1000 (<=0.5USD)	0.5 (0.3–0.9)**	0.9 (0.5–1.6)
	>1000 (>0.5USD)	1.0	1.0
Given food to take to school	Yes	1.5 (1.1–2.2)*	2.0 (0.6–1.5)
	No	1.0	1.0
Given food at school	Yes	6.1 (4.5–8.3)**	6.4 (4.2–9.6)**
	No	1.0	1.0
Drink light sugar soft drinks	Yes	1.6 (1.2–2.1)*	1.0 (0.7–1.5)
	No	1.0	1.0
Dinner time	<=7:00 PM	0.6 (0.4–0.9)**	0.4 (0.5–0.8)**
	>7:00 PM	1.0	1.0
Usually eat breakfast before going to school	Yes	0.9 (0.7–1.3)	0.7 (0.5–0.9)*
	No	1.0	1.0
Drink full sugar soft drinks	Yes	1.0 (0.7–1.4)	
	No	1.0	
Usually eat vegetables	Yes	1.0 (0.4–2.2)	
	No	1.0	
Usually drink fruit juice	Yes	2.0 (0.9–4.0)	
	No	1.0	
Usually eat fruits	Yes	1.5 (0.5–5.0)	
	No	1.0	
Usually eat fried food	Yes	0.7 (0.4–1.2)	0.8 (0.4–1.5)
	No	1.0	
Usually eat starch foods	Yes	0.8 (0.4–1.5)	
	No	1.0	

*p-value < 0.05

**p-value < 0.001

***p-value < 0.0001

Children who usually took food to school were more likely to be overweight or obese than those who did not take food to school (COR = 1.5, 95 % CI = 1.1–2.2, p 0.02). The majority 225 (92.7 %) of the children who took food to school reported taking chips, burgers and doughnuts. Similarly children who were given lunch at school were more likely to be overweight or obese than those who were not given lunch at school (COR = 6.1, 95 % CI = 4.5–8.3, *p* < 0.001). All four private schools that participated in the study provide lunch to their students while public schools do not provide lunch. Food provided to students is mostly starch (Rice, Chips) and protein (Beef, beans) in all the four private schools.

Children who reported often drinking light sugary soft drinks were more likely to be overweight or obese than those who did not report often drinking light sugary soft drinks (COR = 1.6, 95 % CI = 1.2–2.1, p 0.001). Children who reported eating dinner before 7:00 PM were less likely to be overweight or obese than those who reported eating dinner after 7:00 PM (COR = 0.61, 95 % CI = 0.4–0.9, *p* < 0.001). Drinking fruit juice, eating fruits, eating fried food and eating starchy foods were not significant predictors of overweight and obesity as shown in Table 3.

After adjusting for gender, age and school type; eating of breakfast before going to school, and being given money to buy food sold at school was found to be protective against overweight or obesity as shown in Table 3. Provision of lunch at school was found to be a risk factor for overweight or obesity.

## Discussion

This study revealed that 14.7 % of primary school children in Kinondoni district were either overweight or obese. Our findings on prevalence of overweight (10.2 %) concur with findings from a study conducted in 2010 to determine the prevalence of overweight and obesity among children aged 6–12 years in Dodoma and Kinondoni municipalities which reported prevalence of overweight among children in Kinondoni municipality to be 10.7 %. However this study reported the prevalence of obesity for children in Kinondoni municipality to be 11.8 % [15]. Our findings are also comparable to the prevalence of overweight and obesity in some developing and developed countries such as South Africa where 16 % of children aged 6–13 years were reported to be overweight and obese [22], and in Norway where a prevalence of 13.8 % was reported among children aged 2–19 years) [23] and in France with a prevalence of 15.2 % among children aged 3–14 years [24]. This is a serious public health problem given its established link to chronic health problems during childhood and adulthood [25–29].

The high prevalence of overweight and obesity of primary school children in Kinondoni is attributed to factors assessed by our study but also to other determinants of overweight/obesity such as policies that may play a role in childhood obesity such as school policies (e.g., school canteens, play grounds, inclusion of sports hours in school curricular). Other factors include inadequate availability of pavements for pedestrians, paths for bicycling, and public playgrounds for children, which is a big problem for all major cities in Tanzania including Dar es Salaam.

Our study identified several factors related to dietary and behaviour practices to be associated with overweight or obesity. For example, children who walked or used public transport to and from school were less likely to be overweight or obese than those who were driven by private cars or used school buses. Walking or using public transport to and from school is part of being physically active. Children who use public transport normally walk to and from bus stops, which are sometimes at a distance from their homes or schools. Children who are driven by private cars or use schools buses are picked up from home or at school bus stops very close to their homes. Other studies [3–5] conducted in Europe and South America have shown that active form of transport to and from school is associated with decreased risk of overweight and obesity.

Overweight and obesity was also higher among respondents who spent long hours watching television, and playing computer and video games. Studies that examined sedentary habits in children have reported strong association between television watching and obesity [5, 7, 8]. Studies that examined the relationship between the time spent on computers and obesity yielded contradictory findings. For example, a number of studies found no relationship between computer usage and obesity [7, 9–11], while studies undertaken among Portuguese and Brazilian youth found that obesity was related to computer time and not television time in [5, 12].

Children who carried lunch to school; those given lunch at school and those who drank light sugary drinks were likely to be overweight or obese. Children who bought food or drinks at school were less likely to be overweight compared to those who either brought own lunch or ate lunch from school. This could be explained by the fact that most of those who carried money to school were given just enough to buy few snacks and those who were given little amount of money were mostly from families with lower SES and attending public schools. Another possible explanation is that whatever food or drinks bought from school was counteracted by the effect of children being physically active including active mode of transport to and from school. On the other hand children who carried a larger amount of money (>1000 Tshs) (>0.5 USD) to school were more likely to be obese and were mostly attending private schools. This could be due to the fact that food served/sold to children in Private schools mainly consisted of refined carbohydrates with little or no fruits and vegetables. The type of lunch provided coupled with sedentary lifestyle in most private school children could be a reason for the higher prevalence of overweight and obesity among these children.

Our study also revealed that children who often ate breakfast before going to school were less likely to be overweight or obese. This finding confirms the findings of other studies which found that skipping breakfast was associated with obesity among children living in developed [28, 30] and developing [31, 32] countries. Surprisingly, eating fried and starchy foods were found to be associated with decreased risk of obesity. These findings were not anticipated, given that such dietary practices are expected to increase the risk of childhood obesity in developed countries [28]. It is possible that children who are obese reduce their intake of unhealthy foods to cut down weight. Another possible explanation is that overweight or obese respondents may have underreported the types of food so as to paint a picture of consuming a healthy diet.

Children with three or less siblings were at a greater risk of being overweight or obese than those with a higher number of siblings. Similar findings have been reported by another study conducted in Norway [23]. Overweight and obesity was also higher in children who attended private schools compared to those who attended public schools. This could be attributed to the fact that children who attend private schools come from families with higher social economic status than those who go to public schools. Wealthier families can afford to take their children to school using private cars or school buses. They can also afford electronic recreational games such as computer and video games. This has an impact children’s level of physical activity.

There are a number of limitations of this study that need to be pointed out. An obvious limitation is its cross – sectional nature which precludes statements of cause and effect. This becomes especially limiting when interpreting associations that could have two legitimate pathways such as reverse association between obesity and walking to school. Walking to school may have a protective effect on development of obesity or it may simply be that obese children prefer to be driven to school. Also as we pointed out earlier some dietary practices did not give anticipated results. This could be due to the fact that overweight and obese children might be cutting down on unhealthy foods and eating more of health foods to lose weight. Thus findings need to be treated with caution.

Another limitation is that puberty, ethnicity and genetic factors that could have an effect on obesity were not assessed. Given the wide age range of our sample it is likely that puberty and other mentioned factors played a role in the onset of overweight and obesity.

In addition, though questions were designed to be age appropriate we cannot rule out the possibility of recall bias and difficulty in comprehending questions in younger children. To overcome this limitation some of the information provided by much younger children was validated against school records where applicable. Furthermore some younger children failed to answer some of the questions a fact that can have an effect on the measure of association calculated.

Another limitation is that information on the amount of food usually consumed was not collected. Given that staple food for most families in Tanzania is starch (rice, ugali-meal made from maize flour, bananas etc.) this information would have been instrumental in assessing the effect of type of food taken on obesity.

## Conclusion

This study demonstrates that a significant proportion of primary school children in Kinondoni district are either overweight or obese.

Factors related to overweight or obesity were lack of physical activity and unhealthy dietary practices at home and at school. It is therefore recommended that parents and teachers should encourage children to be physically active while at school and at home to reduce rates of obesity in children. Schools/teachers can help children to be physically active by limiting screen time, promoting active transport to and from school and giving preference to outdoor games over video games. Ministry of education needs to formulate/enforce policies that encourage physical activities for school children and regulate quality of foods provided to children at school.
